# Psychological response to the COVID-19 pandemic in Canada: main stressors and assets

**DOI:** 10.1177/17579759211023671

**Published:** 2021-07-16

**Authors:** Mélissa Généreux, Mathieu Roy, Marc D. David, Marie-Ève Carignan, Gabriel Blouin-Genest, S.M. Zeeshan Qadar, Olivier Champagne-Poirier

**Affiliations:** 1 Department of Community Health Sciences, Faculty of Medicine & Health Sciences, Université de Sherbrooke, Sherbrooke, Québec, Canada; 2 Eastern Townships Public Health Department, CIUSSS de l’Estrie-CHUS, Sherbrooke, Québec, Canada; 3 Department of Family Medicine & Emergency Medicine, Faculty of Medicine & Health Sciences, Université de Sherbrooke, Sherbrooke, Québec, Canada; 4 Département de communication, Faculté des Lettres et Sciences Humaines, Université de Sherbrooke, Sherbrooke, Québec, Canada; 5 School of Applied Politics, Faculté des Lettres et Sciences Humaines, Université de Sherbrooke, Sherbrooke, Québec, Canada; 6 National Collaborating Centre for Infectious Diseases, Rady Faculty of Health Sciences, University of Manitoba, Winnipeg, Manitoba

**Keywords:** COVID-19, pandemic, disaster, psychosocial impacts, post-traumatic stress, risk communication, communication strategy

## Abstract

**Background:**

The COVID-19 crisis has unique features that increase the sense of fear, and comes with additional stressors (e.g., confusion, discrimination, quarantine), which can lead to adverse psychological responses. There is however limited understanding of differences between sociocultural contexts in psychological response to pandemics and other disasters.

**Objective:**

To examine how Canadians in different provinces, and with different governance modes and sociocultural contexts, understand and react to the COVID-19 pandemic.

**Methods:**

A web-based survey was conducted from April 8–11, 2020, among a representative sample of 600 Canadian adults from two different contexts (*n*=300 in Quebec, the French part of Canada, and *n*=300 elsewhere in Canada). Two psychological outcomes were assessed: probable post-traumatic stress disorder (PTSD), and probable generalized anxiety disorder (GAD). The roles of various stressors (i.e., threat perceived for oneself or family/friends, quarantine or isolation, financial losses, victims of stigma), assets (i.e., trust in authorities, information received, and compliance with directives) and sources of information used on these two outcomes were also examined. Chi-square tests were performed to examine differences in the distribution of probable PTSD and GAD according to these stressors and assets.

**Results:**

Probable PTSD and GAD were observed in 25.5% and 25.4% of the respondents, respectively. These proportions were significantly lower in Quebec than elsewhere in Canada. Perceiving a high level of threat and being a victim of stigma were positively associated with probable PTSD and GAD (but not quarantine/isolation and financial losses). A high level of trust in authorities was the only asset associated with a lower risk of PTSD or GAD. Interestingly, this asset was more frequently reported in Quebec than elsewhere in Canada.

**Conclusion:**

The COVID-19 pandemic represents a unique opportunity to evaluate the psychosocial impacts on various sociocultural groups and contexts, providing important lessons that could help respond to future disasters.

## Background

Identified for the first time in China in December 2019, the coronavirus disease (COVID-19) was declared a pandemic on March 11, 2020, by the World Health Organization (WHO). COVID-19 rapidly spread globally. Since its first identification among humans, more than 3,091,489 deaths from over 145,834,362 million cases across 219 countries or territories have been reported as of April 23, 2021 (1).

Public health emergencies and disasters, whether naturally occurring or anthropogenic, often affect entire communities. By causing psychological stress, coupled with significant human and material losses and extended social and service disruption, disasters negatively affect the health and well-being of individuals and societies (2). Such traumatic events may result in a wide range of mental health disorders, post-traumatic stress disorder (PTSD) being the most commonly studied (3). A growing body of evidence demonstrates that the prevalence of anxiety, depression, sleep disorders, substance misuse problems, and somatic symptoms are increasing when communities are hit by a disaster (2–6).

As with any other type of natural disasters, the psychosocial risks arising from large-scale outbreaks need to be considered by public health organizations, in conjunction with the risk of infection. The COVID-19 pandemic has unique features that increase the sense of fear (e.g., being transmissible, imminent, invisible, ominously covered by the media) and comes with a number of additional stressors (e.g., mistrust, confusion, misinformation, discrimination (7)). While some fear can stimulate preventive behaviors, extreme fear may lead to adverse psychological responses (8). It thus comes as no surprise that, along with the first wave of the pandemic that has affected the world in the first half of 2020 (and the subsequent second and third wave in 2021), fear has spread as fast as the virus itself, if not faster. Several studies have reported that this fear has led to adverse mental health outcomes (9–11).

Emotional, sociocultural, political, and epidemiological factors all play a vital role in the individual response to stressors (12). For instance, risk information is not received and understood equally. A strengths-based approach, not only focusing on risk factors (or stressors) but also on protective factors (or assets) is important to better understand how mental health and well-being can buffer the adverse effects of psychological stressors (13). This type of research is urgently needed because promoting health and well-being through a salutogenic approach may be just as important as protecting health and safety in a disaster context (14). It has indeed been shown that fostering health assets may complement the usual public health response for people in unfavorable situations (15).

The media also plays a major role in shaping responses to disasters (especially those involving biological threats and risks of contagion). During pandemics, mainstream and social media discourses are often poorly informed by science. This may contribute to public misinformation and misunderstanding, which may fuel a sense of fear and foster a host of adverse psychological responses. There is limited understanding, however, of differences between sociocultural and political contexts in psychological responses to health information (or misinformation). When dealing with pandemics, the authorities’ communication strategies are embedded in multilevel governance (from global to local), which adds another layer of complexity (16). Carrying out more ‘*real-world research*’ is crucial to generate evidence relating to the psychosocial aspects involved during pandemics and how it is shaped by authorities and media discourses under various circumstances (i.e., in different groups or geographic locations (17–19)).

### From the international to the Quebec/Canadian context

Although the COVID-19 pandemic is global and affects more than 219 countries and territories, each country or region has its own epidemiological, demographic, sociocultural, economic, and political specificities, which modulate the psychological response of individuals and communities. In Canada, the COVID pandemic began on January 27, 2020, after an individual who had returned from Wuhan, Hubei, China, tested positive. In the early phases of the pandemic (until the end of February), the spread of the virus was considered ‘*under control*’ by authorities. Until March, all cases were linked to recent travels. The first case of community transmission in Canada was confirmed in the beginning of March.

Even though confirmed cases have been reported in each Canadian province and territory, the province of Quebec (a predominantly French-speaking province), which counts a little less than a quarter of the Canadian population, had more than 50% of all confirmed cases and deaths related to COVID-19 by the end of its first wave of the pandemic (20). Various factors have been underlined to explain this particular epidemiological situation: an aging population living in long-term care facilities, a week of school vacation earlier than elsewhere in the country, and behaviors being potentially less respectful of public health standards due to cultural reasons. Notwithstanding these particularities, the Government of Quebec distinguished itself through its social and political response to the pandemic. Among others, its communication strategy, described by many as being transparent and coherent, while at the same time being reassuring, supportive, and compassionate, was particularly appreciated by Quebecers (21).

### Introduction to the pilot survey

This study looks at how populations with different governance modes and sociocultural contexts understand and react to the COVID-19 pandemic. This is done through a pilot survey conducted among a representative sample of the population living in the province of Quebec and the rest of Canada (ROC). This study aims to 1) capture the psychological response and its associated stressors and assets in the midst of the first wave of the pandemic in Canada, and 2) compare psychological responses, stressors, and assets in Quebec versus the ROC.

## Methods

### Design

This study takes place within a broader research entitled ‘*The role of communication strategies and media discourse in shaping the psychological and behavioral response to the COVID-19 outbreak: an international comparative analysis*’ funded by the Canadian Institutes of Health Research. This multidisciplinary and international research seeks to contribute to a better understanding of how the risk-related information is delivered by authorities and media, and how it is received, understood and used by the public (22). In order to do that, a mixed-method approach was used, including a repeated cross-sectional population-based survey conducted in several jurisdictions, a quantitative and qualitative discourse analysis of the news media and social media, and a network analysis (e.g., information disseminated by the WHO, distribution lists, reception and use of information) to assess how information flows and circulates across levels of governance.

A few weeks after receiving the grant, a questionnaire was built and the conduct of a pilot survey based in Canada was rapidly reviewed and approved by the Research Ethics Board of the CIUSSS de l’Estrie – CHUS. This pilot survey was performed from April 8–11, 2020, among a representative sample of 600 randomly recruited Canadian adults from two different political and sociocultural contexts (*n* = 300 in Quebec, and *n* = 300 in the ROC).

### Recruitment

Any adults (18 years and older) living in Canada and able to answer a questionnaire online were eligible to participate. Recruitment was undertaken by a professional polling firm called Leger 360 (https://leger360.com/). This firm has built an online panel of 400,000 members and has the largest Quebec francophone panel. These members come from several sources (i.e., 50% are recruited randomly by the call center, 35% by invitation and affiliate programs, 5% through social media, 5% by offline recruitment, and 5% through partner programs and campaigns). Target recruitment was carried out to ensure the inclusion of hard-to-reach groups on the internet (e.g., ethnic minorities, young people, seniors) and therefore to increase population coverage and improve the quality of the sample provided. The database is actively managed as follows:

- Interviewers inviting respondents to join the panel, with over 30,000 new panel members recruited per year;
- More than 55,000 respondents per year participating in focus groups who are invited to join the panel;
- By referrals, social media, or to earn extra money, ambassadors can recruit new panel members each day, however each referral is filtered and analyzed;
- Conventional Google ads and website banners.

Participant management for the current study was aligned towards maximizing census representation. The optimal representativeness of the sample was backed by the use of software generating representative samples of the population (i.e., quotas sampling) and by weighting response rates based on age, sex, language, and region.

### Data collection

The questionnaire built for the pilot phase of the survey was available (pre-tested and validated) in English and French. It contained closed-ended questions only and lasted an average of 10–15 minutes. Based on the Knowledge-Attitude-Practice (KAP) model (23), a wide range of aspects were explored, including risk perceptions and beliefs, positive and negative attitudes, and adaptive and maladaptive behaviors. Sociodemographic characteristics (e.g., age, sex, education level) were also assessed.

### Outcomes

Two psychological outcomes were assessed, including probable PTSD and probable generalized anxiety disorder (GAD).

The Primary Care PTSD Screen for DSM-5 (PC-PTSD-5) is a 5-item scale designed for use in primary care settings (24). This scale was designed to identify individuals with probable PTSD. Respondents had to answer five yes/no questions about how the COVID-19 pandemic has affected them over the past month. Those who answered ‘yes’ to at least three of the five questions were considered as having a probable PTSD, based on preliminary results from validation studies suggesting that a cut-off point of three is optimally sensitive (24).

The GAD-7 is based on the diagnostic criteria for GAD described in DSM-IV. This questionnaire is designed for use by health professionals but has also been used in several population-based studies. A composite score ranging between 0 and 21 is possible. A score of 10 or greater indicates a probable GAD that needs to be further evaluated by a clinician (25–27). Using the threshold score of 10, the GAD-7 has a sensitivity of 89% and a specificity of 82% for GAD (28).

### Predictors (stressors and assets)

Various factors that may have influenced the psychological response to the pandemic were considered, particularly those related to information accessibility and the different channels of communication (e.g., traditional, digital, and interpersonal) used and valued (29). The following variables were specifically examined:

- The perception of the level of threat posed by COVID-19 to oneself, and family and friends (very low/low/moderate vs. high/very high).
- Being a victim of stigma or discrimination due to COVID-19 (yes/no).
- Having experienced financial losses of any kind due to COVID-19 (yes/no).
- Having experienced home quarantine or isolation, mandatory or voluntary (yes/no).
- Level of information about the coronavirus, with a scale ranging from 1 to 10 (high [9–10] vs. lower levels [0–8]).
- Level of trust in public authorities, with a scale ranging from 1 to 10 (high [9–10] vs. lower levels [0–8]).
- Level of compliance with the directives given by the authorities, with a scale ranging from 1 to 10 (high [9–10] vs. lower levels [0–8]).

In addition to these stressors and assets, the sources used to get informed about COVID-19 were examined, some of them hypothesized to act as psychological stressors (e.g., social networks) and others as assets (e.g., authorities). Respondents had to report the frequency of use, which was subsequently dichotomized as ‘a lot/somewhat’ vs. ‘not much/not at all’, for each of the following sources of information: WHO; Canadian federal government; provincial government; public health authorities; health professionals; news media (television, radio, newspapers); friends, family, and co-workers; social networks; the Internet.

### Data analysis

Data were weighted for age, sex, and regions in each province to ensure the representativeness of the sample. Statistical analyses were performed (chi-square tests) to compare various key variables (i.e., psychological outcomes, stressors, assets) between subgroups (i.e., Quebec vs. the ROC, men vs. women, young vs. older adults, low vs. higher education level) and to assess the relationships between the set of stressors and assets, and the psychological outcomes under investigation.

## Results

The distribution of stressors, assets, and sources of information in Quebec versus elsewhere in Canada are displayed in Table 1. Overall, these data suggest that during the first wave of the pandemic in Canada, home quarantine or isolation, as well as financial losses, were the norm for most Canadians, either in or outside the province of Quebec, even though Quebecers appeared more likely to apply home isolation measures (88.6% vs. 72.8%, *p*< 0.05). One of the most salient differences observed between the province of Quebec and the ROC regards the level of trust in authorities, with about half of Quebecers reporting a very high degree of confidence in public authorities (49.6%), compared to 26.8% for those in the ROC. More Quebecers felt they have the information they need to understand the coronavirus fully (83.7%) compared to respondents from the rest of the country (60.8%). They were also more inclined to cite provincial government and television as a regular source of information, while more Canadians outside this province privileged the federal government to get informed about the coronavirus.

**Table 1.** Distribution of stressors, assets, and sources of information in Quebec versus in the rest of Canada (ROC).

	*Quebec n* = 300 (%)	*ROC n* = 300 (%)	*Total n* = 600 (%)
Stressors			
Threat to oneself perceived as high	25.7	23.0	23.6
Threat to family or friends perceived as high	27.9	25.6	26.1
Home quarantine or isolation	88.6*	72.8*	76.5
Financial losses	53.2	61.0	59.1
Victim of stigma or discrimination	7.5	13.2	11.9
Assets			
High level of information	44.0	39.6	40.6
High level of trust in public authorities	49.6*	26.8*	32.2
High level of compliance with the directives	77.3*	64.3*	67.4
Sources of information used			
WHO	48.2*	66.8*	62.4
Federal government	65.0*	87.1*	81.9
Provincial government	94.3*	88.2*	89.6
Public health authorities	83.3	84.8	84.5
Health professionals	67.4*	78.8*	76.1
Media (television)	80.0*	69.4*	71.9
Media (radio)	40.9	43.6	42.9
Media (newspapers)	38.0	34.1	35.0
Friends, family, or co-workers	42.8	50.0	48.3
Social networks	45.0*	34.7*	37.2
Internet	70.7	65.4	66.7

* *p* values from the Chi-square tests < 0.05.

Overall, probable PTSD and GAD were observed in 25.5% and 25.4% of the Canadian respondents, respectively. However, probable post-traumatic stress related to the pandemic was more frequent outside Quebec (27.5%) than inside the province (18.8%). The same is true for generalized anxiety (28.8% outside Quebec versus 14.2% inside the province). Noticeable differences in psychological responses were also found between men and women, and according to age groups, with men and older people being less likely to suffer from post-traumatic stress related to the pandemic and generalized anxiety than women and younger adults, respectively (Table 2).

**Table 2.** Psychological outcomes according to sociodemographic characteristics.

*Sociodemographic characteristics*	*Probable PTSD (%)*	*Possible GAD (%)*
Province		
Quebec	18.8*	14.2*
Rest of Canada	27.5*	28.8*
Gender		
Women	30.7*	26.6
Men	19.8*	24.1
Age		
18–44 years	31.8*	32.0*
45–64 years	23.8*	22.1*
65 years or more	16.8*	18.3*
Highest level of education		
High school or less	25.2	21.3*
College	28.9	31.3*
University	19.6	21.3*
Total	25.5	25.4

* *p* values from the Chi-square tests < 0.05.

As shown in Table 3, psychological stressors that were significantly associated with either probable PTSD or GAD relate to stigma and to the fact that the pandemic is perceived as a high threat to individuals and loved ones (family or friends) while, surprisingly, this was not the case for home quarantine/isolation and financial losses. On the opposite side, respondents who were more trustful of authorities seemed less likely to report PTSD and GAD symptoms than others. In addition, probable PTSD and GAD were found to be statistically more frequent among respondents who reported regularly using the WHO or federal government as a source of information, while this was not the case for sources of information at the provincial government level (Table 3). Except for the provincial government, most sources of information, including the news and social media, were associated with a greater risk of probable PTSD or GAD.

**Table 3.** Psychological outcomes according to stressors, assets, and sources of information to get informed about COVID-19.

	*Probable PTSD (%)*		*Probable GAD (%)*	
	Presence of stressor (%)	Absence of stressor (%)	Presence of stressor (%)	Absence of stressor (%)
Threat to oneself perceived as high	34.8*	22.9*	43.6*	19.7*
Threat to family or friends perceived as high	33.3*	22.9*	37.7*	21.2*
Home quarantine or isolation	24.8	27.3	26.9	20.3
Financial losses	27.0	24.3	28.4	22.6
Victim of stigma or discrimination	46.8*	22.2*	43.5*	23.2*
	Presence of asset (%)	Absence of asset (%)	Presence of asset (%)	Absence of asset (%)
High level of information	26.2	25.0	23.9	26.3
High level of trust in public authorities	19.7*	28.4*	17.6*	29.2*
High level of compliance with the directives	28.9*	18.1*	23.8	28.6
	Source used (%)	Source not used (%)	Source used (%)	Source not used (%)
WHO	29.6*	18.6*	27.0	18.5
Federal government	28.1*	13.4*	26.8	19.3
Provincial government	26.3	18.2	23.9*	38.7*
Public health authorities	27.1*	16.3*	21.9	16.1
Health professionals	28.3*	16.0*	28.8*	15.6*
Media (television)	27.0	20.0	26.2	24.0
Media (radio)	30.7*	21.1*	31.0*	22.1*
Media (newspapers)	35.6*	20.2*	31.1*	22.8*
Friends, family, or co-workers	28.2	23.2	30.3*	21.1*
Social networks	30.3*	22.7*	30.1*	22.0*
Internet	27.7	20.9	28.2*	20.1*

* *p* values from the Chi-square tests < 0.05.

## Discussion

By looking at the psychosocial impacts of COVID-19, this Canadian survey has provided clear initial results: less than a month after being declared a pandemic, COVID-19 was wreaking havoc in Canada, with one quarter of respondents showing significant symptoms of post-traumatic stress and generalized anxiety. Similar findings have been observed in another national survey conducted in Canada from May 8–12, 2020 (30), where 25.5% of respondents indicated moderate to severe anxiety levels using the GAD-7 scale. This survey also found, just as we did, that women and younger adults were more likely to feel anxiety during the pandemic.

According to the ‘pre-pandemic’ literature, it is estimated that 2.5% to 5.0% of adults generally present symptoms compatible with generalized anxiety (26, 31, 32). It should, however, be noted that different scales have been used to measure GAD and that most studies were conducted many years before the pandemic. Based on our findings, the current level of GAD in Canada (25.4%) is considerably higher than before the pandemic. As a comparison, the estimated prevalence of GAD among Canadian adults during wave 1 of the pandemic was similar, if not higher, to that observed in Fort McMurray six months after the devastating 2016 wildfires, where the one-month prevalence of GAD, measured via the GAD-7, was 19.8% (33). International studies also showed that emotional distress and psychopathological disorders have exploded since the onset of the COVID-19 pandemic. In the United States, around one third of the population reported depression or anxiety symptoms since the beginning of the crisis (34). The main reasons advanced for this adverse psychological response are economic concerns, health and safety implications, and social distancing measures (34). Studies from Italy and Belgium found that lockdown delayed sleep timing, increased time spent in bed, and impaired sleep quality (35). A study in China also reported COVID-19-related increase in anxiety, especially among younger people (<25 years; 36).

The global crisis is clearly having an impact on wellness. In some places, however, people may be better protected psychologically. This seems to be the case in Quebec. Interestingly, this province was by far the most affected by the virus spread and presented the highest morbidity and mortality during the first wave. This suggests that the epidemiological situation of COVID-19 over a given territory is not the only factor that can trigger psychological problems. In this regard, findings emerging from this survey are very instructive on how information disseminated from the global, national, and sub-national levels, as well as how it is received and understood by the public from various sociocultural contexts, may positively or negatively affect the psychological response to major health threats. A high level of trust in authorities was associated with a lower risk of probable PTSD or GAD. Since this asset was more frequently reported in Quebec than elsewhere in Canada, it suggests that the more favorable situation observed in Quebec (in terms of psychological response) may be partially explained by a greater trust in the information received, no matter the impacts of the pandemic in terms of number of cases and mortality rate. This should convince practitioners and decision-makers that fundamental and often underestimated assets are available at the population level and that mobilizing such assets may buffer the adverse effects of pandemic-related stressors on mental health. As a complement to their tremendous efforts to fight the biological threat posed by COVID-19, public health authorities should invest more in a salutogenic approach aimed at fostering assets that create health, in an attempt to restore balance in health promotion and protection.

Respondents in Quebec were also less likely to rely on the WHO or federal government (and more likely to rely on their provincial government) as their regular source of information, which may also explain some of the psychological differences observed between Quebec and the ROC. One possible explanation to support these findings would be that information not translated into the main spoken language (i.e., French), and not sufficiently tailored to the local culture, may fuel confusion, misunderstandings, and worries, while more accessible and contextualized information may promote a sense of security (37). Such ‘*personalized*’ communication strategies (i.e., daily press conferences given by governmental and public health authorities at the provincial level) seemed to be very effective among people in this province.

Economic impacts following the COVID-19 pandemic varied widely and each province, economic sector, and population was affected in different ways, some even benefiting from the unusual situation (e.g., e-business, delivery, green tech, construction). The pandemic, causing notably a recession in Canada, is nonetheless an important potential source of stress and anxiety for Canadians. Being equally affected at the economic level compared with other provinces, Quebec presents, however, interesting economic data that, combined with our results showing that Quebecers had a greater confidence in public authorities, may help to alleviate the mental health impacts of the pandemic through the appearance of economic control, explaining our result that financial loss is less of a stressor for the province of Quebec (38).

Although informative on the potential factors that may influence psychological response in times of a pandemic, this study also has several limitations that must be underlined. First, its cross-sectional nature precludes the inference of causality between stressors/assets and psychological outcomes. Second, the way data were collected (through an online questionnaire) may have somewhat impaired the representativeness of the sample, with adults who cannot read and those who are less comfortable using a computer being potentially underrepresented. Finally, our study is based on self-reported measures which may be subject to information bias. While the GAD symptoms were assessed using a previously validated scale with good psychometric properties, PTSD symptoms were measured via a newer scale that should be further validated. In the same vein, most measures related to information accessibility and the different channels of communication used and valued were developed by our research team for the current study.

## Conclusion

These early findings strongly suggest that the pandemic has had a significant psychological impact on Canadians. It raises how multilevel communication strategies are key during a health emergency and how information disseminated in media and other networks impact health and well-being. Refining our understanding of how various groups of the population perceive risks and react to them is not only vital to improve risk communication strategies, but also to mobilize assets within communities in order to tailor public health action during and after pandemics (or other disasters). This is especially important as actors from multiple sectors and at multiple levels need to develop a common vision and combine their efforts in finding solutions to minimize health burdens caused by these catastrophic events.

It will be crucial to monitor how psychological responses change over time and to adapt available support accordingly. This unique survey, leveraging on an interdisciplinary approach, was the first of a series of three population-based surveys to be conducted in different countries (22). With larger samples from Canada and other countries, our future international surveys will not only allow monitoring of trends in the psychosocial impacts of the pandemic, but also comparison of these outcomes across countries with different sociopolitical and institutional backgrounds.
